# Three Weeks in an Eye Hospital

**Published:** 1920-08-28

**Authors:** 


### FROM THE PATIENT'S POINT OF VIEW.

### Three Weeks in an Eye Hospital.

It is not too much to say that during the past six eventful years 10 per cent. of the male population of Great Britain has been treated in hospital; and the point of view herein expressed is amplified by the writer having spent —in irregular instalments—nearly two years in various hospitals trying to get well. My latest experiences follow my admission to one of the well-known hospitals because of a troublesome eye, said to be largely due to constitution, which seemingly acts in an inconsistent manner from time to time. It is on these occasions one is forced to assume their eyes have been a source of discomfort to some persons ever since their world began.

But the old-fashioned prejudice in regard to entering hospital is now broken down. Nowadays no one seriously believes that a surgeon will take an eye out, scrape it, and, after untold agony to the patient, replace it; or that eyes are needlessly enucleated or removed; an empty eye-socket offers little in the way of experience to anyone except its owner. We live in enlightened times, and even the doctor's best Latin—used in the worst cases—is not absolutely foreign to the patient, who, should he be blessed with a retentive memory, benefits from the comments made by the doctor on his condition. The various hieroglyphics transcribed on his case-board at the head of the patient's bed are, also, no longer Greek to him. In this hospital a fair-haired youngster, aged *five*, gravely stated at the dinner-table that the doctor said "his brother had *gli'oma*, but he (the youngster) was sure it wasn't *that*!"

I found that various members of the hospital staff had grown old along with me, and, as the external treatment of the patient is to some extent augmented by the kindly personal interest of the nurses, my improvement was rapid. A fellow-patient found, however, that, although seven years previously he had been able to see from the hospital windows the buildings on the opposite side of the street, that privilege, on this return for treatment, was denied him. Like Vespasian, I should prefer to die standing; anyway, I do not wish to die in hospital. If this did occur during one of my periodic visits the history of my decease could be chronicled somewhat as follows: The sister startles the newest probationer with the casual comment that "Blank (referring to myself) will probably die to-night." The newest probationer then excitedly repeats to the next ditto the possibility of my soon being interested in descriptions other than earthly. The next probationer discusses in an undertone with the staff nurse various delightful etceteras in the gentle art of laying me out decently. The staff nurse next considers it nearly time I showed less signs of vitality and proceeds to gather together screens from the four corners of the ward, arranging them in rhythmic folds around my couch; in the meantime, I begin to sit up and take notice whilst she sends for a stethoscope and/or stenographer. The sister is pleased to learn from the staff nurse that what was once merely a casual diagnosis. in the opening sentence of this series of events, is gathering credence—and she sends for the matron. The matron asks me if I am comfortable, to which I reply, with thanks, as firmly as the sad circumstances admit. She, in turn, then sends for the house surgeon, who, thank heaven! is out—for it is only that knowledge which fortifies my decision to remain alive! But the house surgeon returns, and, after viewing me with grave suspicion, he sends for the physician. An hour later the early-morning activity in the wards commences and the screens are replaced in their respective corners. . . . Am I characterised as "The Man Who Came Back"?

No, I should not care to die in hospital; yet I cannot express completely enough my admiration for those who so splendidly carry on our hospitals, their self-less devotion to duty, irksome as it must sometimes be. While it is common knowledge that the emoluments attached to the nursing profession are—in proportion to present-day values —ridiculously small, it is sometimes overlooked how unusually long hours are called for. The Daylight Saving Bill was in operation here years before Mr. Willett anticipated getting it through the House of Commons; and only direct action of the right description is entertained in hospital, for the amelioration of suffering mankind.

The patient sees the professional side chiefly of his nurse. For all that, he refuses to believe she can be anything but intensely human. So it is not untoward to opine that if, in hospital, every effort must be made to prevent breaches in etiquette that, when not on duty, she may then pay attention to etiquette in breeches! H. L.

